# The Full Lower Denture and the Substitute Offered*Read before the National Society of Denture Prosthetists, Milwaukee, Wisconsin, August 1-13, 1921. Reprinted from *Journal N. D. A*., March, 1922.

**Published:** 1922-05

**Authors:** Elbert Britten Owen

**Affiliations:** St. Louis


### THE FULL LOWER DENTURE AND THE SUBSTITUTE OFFERED*

. BY ELBERT BRITTEN OWEN, D.D.S., ST. LOUIS

For a number of years I have been of the opinion that some of the principles incorporated in denture construction were erroneous, and several years of exclusive practice in constructing dentures and observing the effects of their use has proved conclusively to me that certain practices are harmful, and, if followed persistently, are actually disastrous to the foundation for artificial dentures. I have, therefore, reason .to believe that these principles are erroneous and unscientific.

### STATEMENT OF ERRONEOUS PRINCIPLES

My efforts to secure such a strong adhesion in lower dentures that they could be removed only with difficulty has caused me more trouble than any other part of my work. In the earlier years of my practice as a specialist I held that condition to be an ideal desideratum in a lower denture, and worked faithfully to that end, but the only cases handled in such manner that have been satisfactory are those cases which were constructed at clinics, and which I never saw again after having placed the dentures in the mouth.

Extreme adhesion is never retained and is not desirable on the lower jaw, as it is too severe a strain on the small area of surface covered. Such adhesion is obtained only by extreme tissue compression, which the tissues resent, causing unnecessary resorption, atrophy of the soft tissues, and ultimate bone tissue destruction.

Border compression is harmful, and is even worse than total compression, as it reduces the area of compression;

* Read before the National Society of Denture Prosthetists, Milwaukee, Wisconsin, August 1-13, 1921. Reprinted from *Journal N. D. A.*, March, 1922.

and the smaller the area, the sooner destructive change is caused in the tissues. This square area comparison accounts for the fact that strong adhesion obtained through compression on the upper jaw will last longer than on the lower jaw. Border compression is permissible, however, on the posterior margins of upper dentures, provided it is done with moderation; but this procedure, commonly known as postdaming, has been largely overdone. In mandibular cases, where submucous tissue is missing and the bony structure is covered only by a thin, tightly drawn mucous membrane, this compression is positively injurious. In practicing this method of tissue compression, one is compelled to be constantly making adjustments, changing the border, and eventually, when the tissue has been absorbed by the compression, the denture becomes loose, the patient is dissatisfied, and the operator is compelled to resort to the practice of "bushing" dentures and "chasing" tissues. By "chasing" tissues I mean the continued following up of changes which take place in the mouth, to be treated in the same manner as when this change occurred in the first place. I do not believe it is necessary to be constantly refitting dentures, and I feel that it should not be done; especially should it not be done in mandibular cases. By this assertion I do not mean to condemn entirely the practice of refitting dentures, as this procedure is sometimes necessary in treatment cases to serve the patient until a time when so-called permanent dentures may be constructed; but it is of little use in the case of permanent dentures to continually follow up this procedure merely to secure renewed adhesion for it will gradually destroy what foundation is left for a denture. Nothing in the foregoing statement should, however, be construed as opposing tissue placement under certain conditions.

Another erroneous method of securing adhesion is the extension of the border of the denture beyond the point where it can be endured permanently, and especially is this true of the lower lingual cuspid region and the mylohyoid region. This extension, when carried beyond a point of endurance, must be trimmed off from time to time, causing annoyance and expense to both patient and operator, and then such retention as was previously gained through this undue extension has been lost. The normal structures of the mouth, particularly of the lower jaw, should be only slightly displaced, and the impression technic should be designed to meet this condition.

The position I take is one opposed to extreme adhesion in the lower denture and the two methods used in securing it, viz., compression of tissues and undue marginal extension. It will be understood that adaptation without compression, plus proper extension, will almost invariably give some adhesion, most of which is retained indefinitely if the occlusion is kept balanced, and in such case very few marginal adjustments are necessary. In many cases where dentures have been worn for years, sometimes from twenty-five to thirty years, with no apparent tissue change and no inflammation, there is seldom very much, if any actual adhesion, and while these old-time dentures are not by any means of ideal construction, lacking in many instances both adaptation and extension, as well as occlusion, I nevertheless believe they are better dentures than some that are constructed today, because they present no destructive process of the tissues. I believe it is possible—in fact, I know it is not only possible, but practicable—to construct lower dentures that are comfortable and efficient, that maintain stability during the legitimate uses that throw the greatest strain upon them, and that do not have to be constantly trimmed and adjusted. I can make this statement more definite by saying that it should be necessary for but fifty per cent. of the lower dentures to be touched after being placed in the mouth, and in the other fifty per cent. the adjustments should be reduced to the minimum. The extension and border adjustment which make this result possible are governed and controlled by the impression technic.

### WHAT SHOULD BE EXPECTED OF A LOWER DENTURE

A lower denture should maintain stability during all legitimate usage, in connection with the position of the structures, which offers the greatest resistance to stability. The denture will, in most instances, possess moderate adhesion, and should be worn with comfort and without having to be constantly altered and adjusted.

### STATEMENT OF FUNDAMENTAL PRINCIPLES

The following principles are involved in producing the desired results: adaptation, extension, border adjustment, little or no displacement of tissue, favorable application of leverage arch formation, occlusion and articulation. These fundamental principles are so thoroughly understood that they need no particular discussion, except to add that they must be incorporated in every denture constructed and by a technic so simple that anyone of ordinary skill may make use of it.

Definite control of the necessary material is the most important factor in the technic. Some of the greatest difficulties in the past have been due to a lack of such control and in working with materials which do not possess the properties of enabling the operator to accomplish the desired results.

Modeling compound possesses properties that, when used alone, are very difficult to control to a degree of accuracy that is required in a finished impression. A high degree of skill is required to manipulate it, and even with that accomplishment I doubt if it is ever manipulated accurately. This latter statement is made with due regard for the great skill of some modeling compound users. Modeling compound, however, does possess some properties which are desirable in the process of making a complete impression.

Plaster of Paris, when used alone, will reproduce accurately the surface it covers, and, if applied at the proper consistency, will not unduly compress or displace tissue. When used alone, however, it is almost impossible to control, as it is difficult to place the plaster in the desired area and make an impression that will definitely outline the denture. This difficulty, however, can be overcome by a technic in which both modeling compound and plaster are used. In the earlier methods of using a modeling compound tray, which was trimmed indiscriminately with a knife, according to the operator's judgment, and was followed by filling the tray with plaster and flowing it in the mouth, there was not sufficient accuracy, as there was no means of determining just how far the operator should seat the impression in order to cover the exact area desired. If the impression were seated entirely, the margins of the impression would, as a general rule, be too long, as the margins of the compound tray had been formed by guess. Even with the later technic of adjusting the margins of the modeling compound tray by means of muscle trimming, the impression, in the majority of cases, was not correct, as the operator could not determine just when sufficient muscle trimming had been done. It is obvious that if the modeling compound tray is accurately muscle trimmed, the tray can be seated with the soft plaster until it offers resistance, which will mean definite control in this step.

The position of the oral structures that has the greatest tendency to dislodge a lower denture is, from a buccal and labial aspect, when the mouth is opened rather wide, and from a lingual aspect when the tongue is protruded rather well out of the mouth to represent the legitimate movement of this organ in casting food from one side of the mouth to the other. If a modeling compound tray that does not possess perfect adaptation, and consequently has no special molecular adhesion, will remain seated on the lower jaw when the mouth is open or the tongue protruded, or when both these conditions prevail, it is sufficient evidence that the margins of the tray are not too long. This is the test to which the modeling compound tray is submitted, and it must withstand this test before any attempt is made to use it in floating plaster over the jaw.

TECHNIC BRIEFLY STATED

It has always been my intention to follow a technic covering the principles involved that does not require much time and gives such control as will not make repeated and continued efforts necessary in order to secure a satisfactory impression. The first step in this procedure is a snap impression. To this little attention is given, other than to make certain that the impression covers more area in every direction than the operator hopes to cover with the finished denture. This means, of course, that the snap impression, which is taken in modeling compound with any ordinary lower tray, will, as a general rule, considerably displace and distend the structures. For this snap impression Kerr's, or some other similar compound which becomes plastic at a lower temperature than the S. S. White tray compound, is used. This snap impression is removed from the metal tray, and the excess, particularly in thickness, is cut away when the operator is ready for the muscle trimming process. I call this procedure "muscle trimming" for the lack of a better term, although it is not done for the purpose of definitely establishing muscle position in the margin of the modeling compound, because, if such were the case, this definite marginal adjustment would be obliterated by the application of the plaster that is to follow. The system of muscle trimming is not very, if any, different from that advocated by the Greene Brothers many years ago, and is used for the purpose of reducing the margins of the tray to a point where they are not too long. When this process of muscle trimming, which is done a section at a time, usually requiring from four to six sections with which to complete the buccal and lingual borders, has been finished the tray is ready for the test to determine if the muscle trimming has been properly done, or if the margins have been reduced to a length which is not too great. This first test is to place the tray in the mouth, hold it in position with the thumb and forefinger of each hand, have the patient open the mouth rather wide, remove the thumb and forefingers from contact with the tray, and observe if the tray is lifted from its position. If the tray is lifted from its position, the buccal and labial margins are too long, and the process of muscle trimming must be repeated until the tray will remain in position. The second test is made with the tongue protruded. If, when holding the tray as before stated, the tongue is protruded and the pressure is released from the tray, it is lifted from its position by lingual attachments, then in that case the lingual margins are too long, and the muscle trimming process in this region must be repeated until it will withstand the test without the tray being lifted from position. Having thus adjusted the margins of the tray, the remaining part of the procedure is simple. A mixture of plaster is made, rather thin and quick setting, accelerated with a saturated solution of potassium sulphate. The compound tray is filled with this thin mixture of plaster and carried into the mouth, when the plaster is shaken from the tray, which is followed by a slow, but complete seating of the tray without much pressure. The seating of the tray can be determined by the resistance that is offered. The mouth is then opened wide enough to produce some tension of the buccal and labial structures, and the tongue is protruded to a moderate degree. It is permissible at this point, if necessary, to assist the cheeks to a position of rest under these conditions. The tray and plaster are held in position until the plaster has crystallized. On removing the impression from the mouth, it will be found that the layer of plaster is thin, but uniform, and, as a rule, the margins will be completely covered with plaster. This impression will definitely outline the finished denture, and in fifty per cent. of the cases this finished denture will not have to be altered.

